# Supplementary figures and images for: Lactate Can Modulate the Antineoplastic Effects of Doxorubicin and Relieve the Drug’s Oxidative Damage on Cardiomyocytes
Source: Cancers (Basel). 2023 Jul 22;15(14):3728. doi: 10.3390/cancers15143728 (PMC10378253; doi:10.3390/cancers15143728)

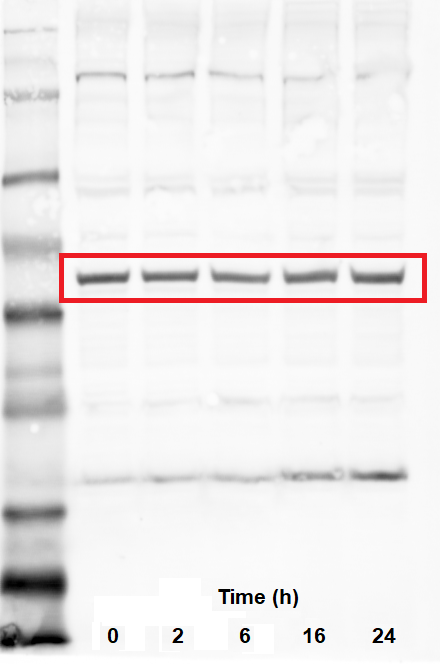

Supplement: Supplementary file 1 [file cancers-15-03728-s001.zip › REVISED Uncropped WB Images/Figure 1/CaCo2/CaCo2 doxo 1uM ACTIN.tif]

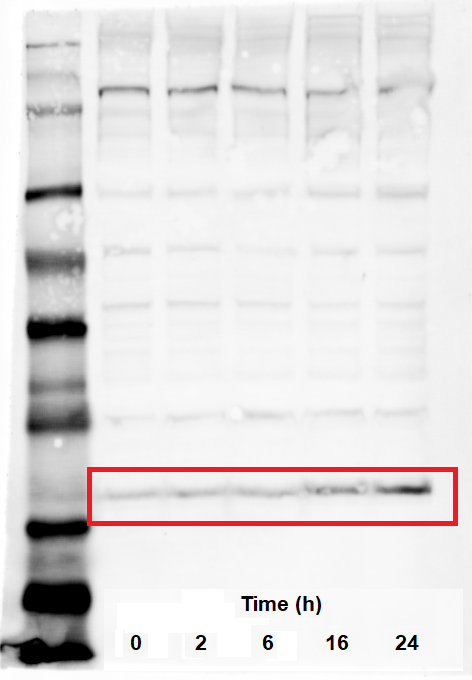

Supplement: Supplementary file 1 [file cancers-15-03728-s001.zip › REVISED Uncropped WB Images/Figure 1/CaCo2/Caco2 doxo 1uM H2AX.tif]

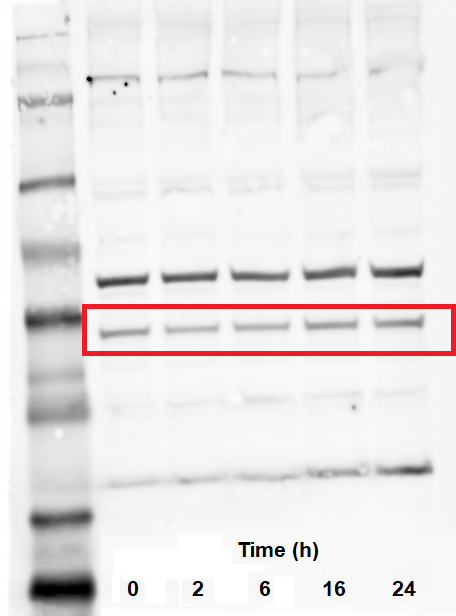

Supplement: Supplementary file 1 [file cancers-15-03728-s001.zip › REVISED Uncropped WB Images/Figure 1/CaCo2/CaCo2 doxo 1uM LDHA.tif]

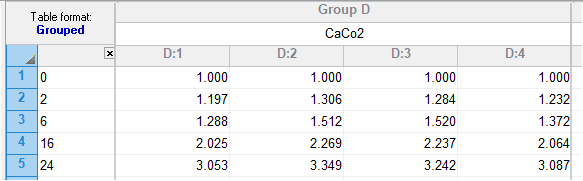

Supplement: Supplementary file 1 [file cancers-15-03728-s001.zip › REVISED Uncropped WB Images/Figure 1/CaCo2/Densitometry H2AX in CaCo2.tif]

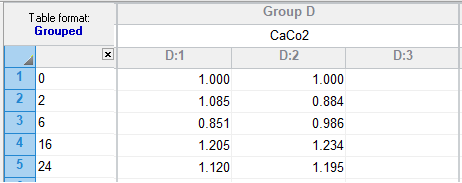

Supplement: Supplementary file 1 [file cancers-15-03728-s001.zip › REVISED Uncropped WB Images/Figure 1/CaCo2/Densitometry LDHA in CaCo2.tif]

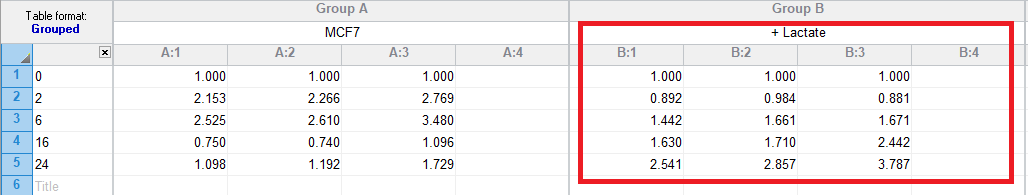

Supplement: Supplementary file 1 [file cancers-15-03728-s001.zip › REVISED Uncropped WB Images/Figure 1/MCF7 LACTATE/Densitometry H2AX in MCF7.tif]

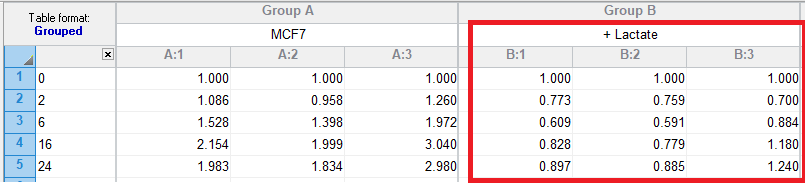

Supplement: Supplementary file 1 [file cancers-15-03728-s001.zip › REVISED Uncropped WB Images/Figure 1/MCF7 LACTATE/Densitometry LDHA in MCF7.tif]

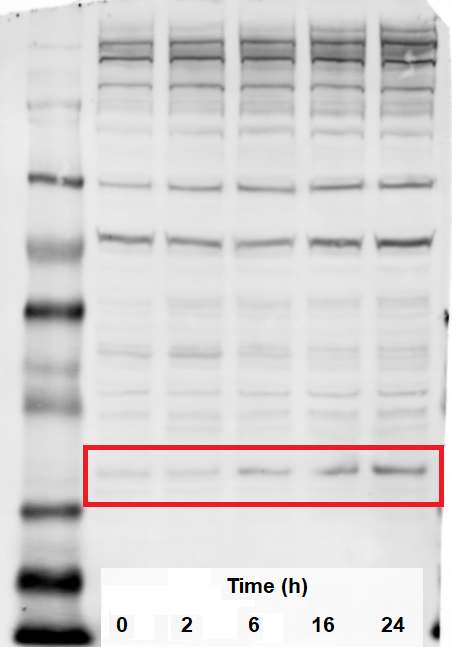

Supplement: Supplementary file 1 [file cancers-15-03728-s001.zip › REVISED Uncropped WB Images/Figure 1/MCF7 LACTATE/MCF7 LACT 20 mM H2AX.tif]

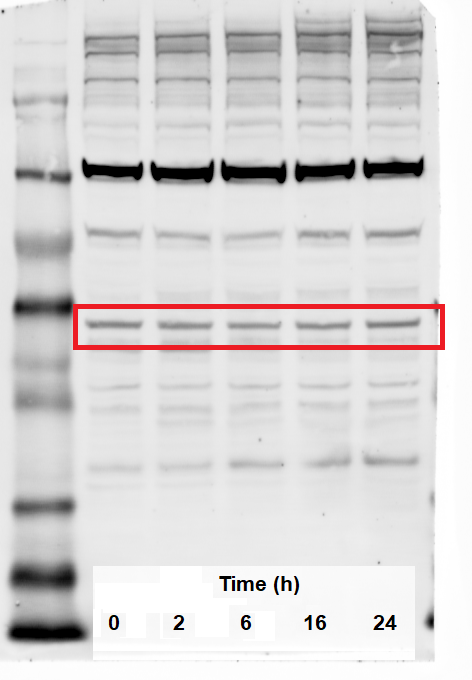

Supplement: Supplementary file 1 [file cancers-15-03728-s001.zip › REVISED Uncropped WB Images/Figure 1/MCF7 LACTATE/MCf7 LACT 20mM LDHA.tif]

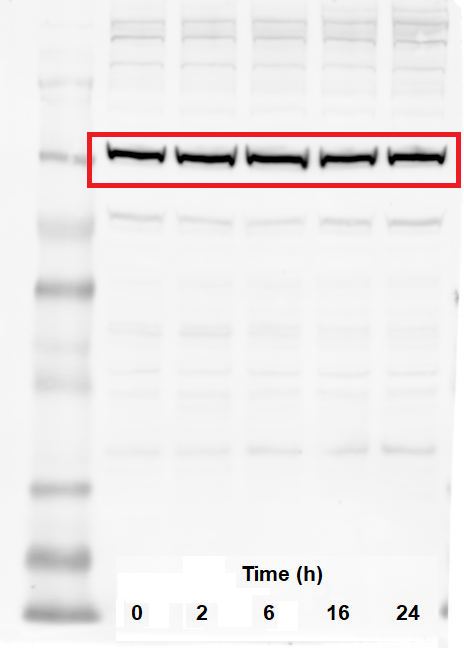

Supplement: Supplementary file 1 [file cancers-15-03728-s001.zip › REVISED Uncropped WB Images/Figure 1/MCF7 LACTATE/MCF7 LACT doxo HSC70 internal control.tif]

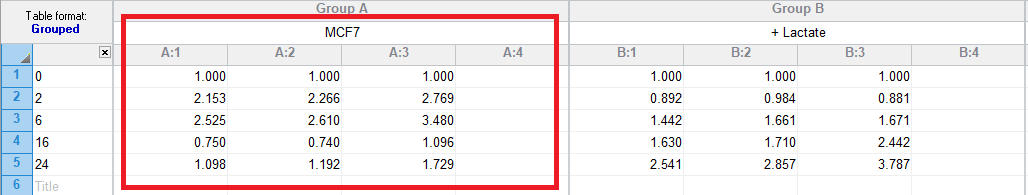

Supplement: Supplementary file 1 [file cancers-15-03728-s001.zip › REVISED Uncropped WB Images/Figure 1/MCF7/Densitometry H2AX in MCF7.tif]

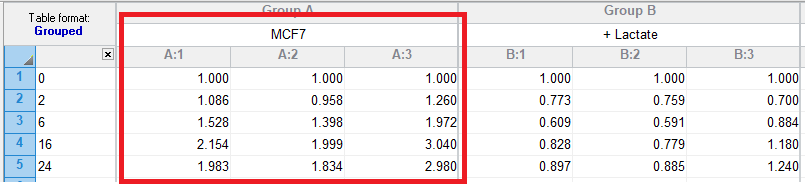

Supplement: Supplementary file 1 [file cancers-15-03728-s001.zip › REVISED Uncropped WB Images/Figure 1/MCF7/Densitometry LDHA in MCF7.tif]

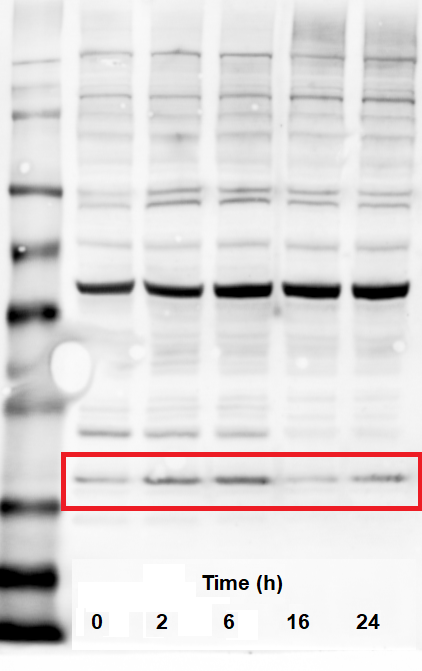

Supplement: Supplementary file 1 [file cancers-15-03728-s001.zip › REVISED Uncropped WB Images/Figure 1/MCF7/MCF DOXO H2AX.tif]

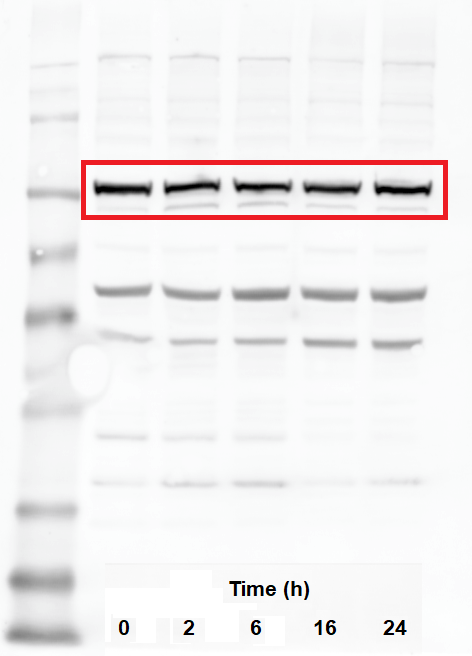

Supplement: Supplementary file 1 [file cancers-15-03728-s001.zip › REVISED Uncropped WB Images/Figure 1/MCF7/MCF7 DOXO HSC70 internal control.tif]

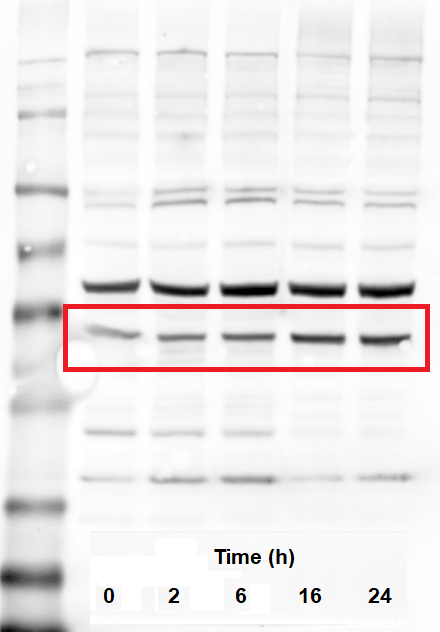

Supplement: Supplementary file 1 [file cancers-15-03728-s001.zip › REVISED Uncropped WB Images/Figure 1/MCF7/MCF7 DOXO LDHA.tif]

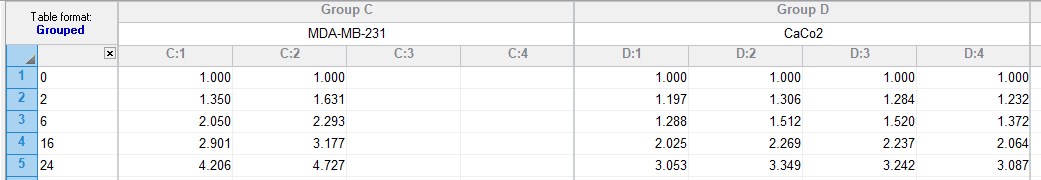

Supplement: Supplementary file 1 [file cancers-15-03728-s001.zip › REVISED Uncropped WB Images/Figure 1/MDA-MB-231/Densitometry H2AX in MDA-MB-231.tif]

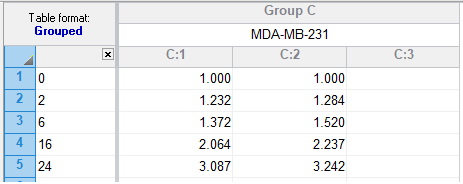

Supplement: Supplementary file 1 [file cancers-15-03728-s001.zip › REVISED Uncropped WB Images/Figure 1/MDA-MB-231/Densitometry LDHA in MDA-MB-231.tif]

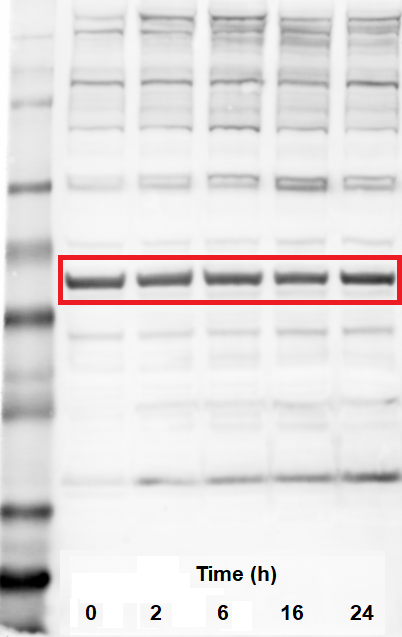

Supplement: Supplementary file 1 [file cancers-15-03728-s001.zip › REVISED Uncropped WB Images/Figure 1/MDA-MB-231/MDA-MB-231 doxo ACTIN.tif]

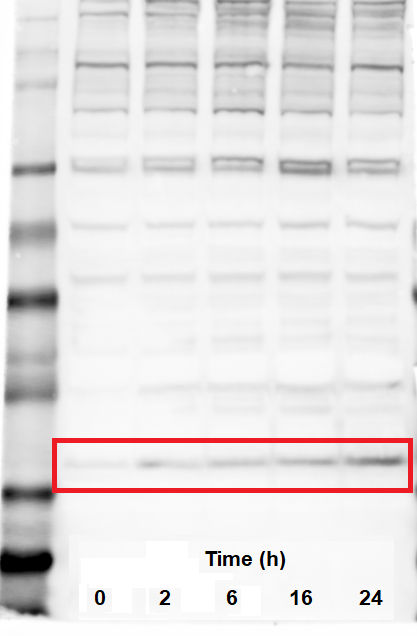

Supplement: Supplementary file 1 [file cancers-15-03728-s001.zip › REVISED Uncropped WB Images/Figure 1/MDA-MB-231/MDA-MB-231 doxo H2AX.tif]

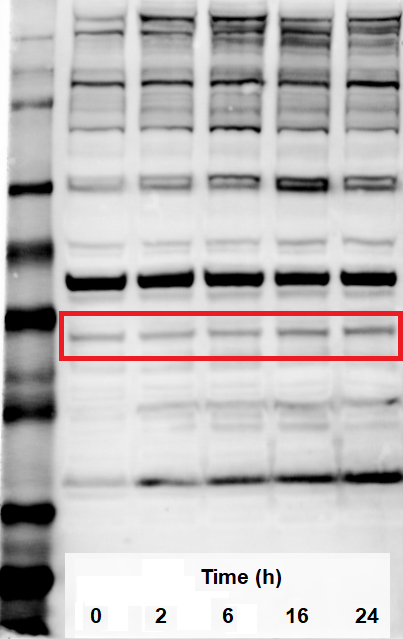

Supplement: Supplementary file 1 [file cancers-15-03728-s001.zip › REVISED Uncropped WB Images/Figure 1/MDA-MB-231/MDA-MB-231 doxo LDHA.tif]

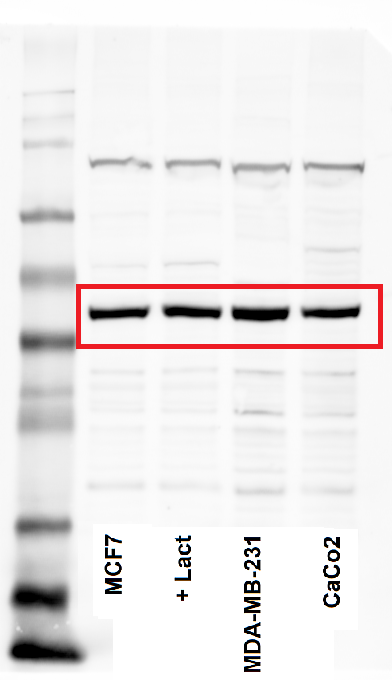

Supplement: Supplementary file 1 [file cancers-15-03728-s001.zip › REVISED Uncropped WB Images/Figure 3/4 cell lines ACTIN.tif]

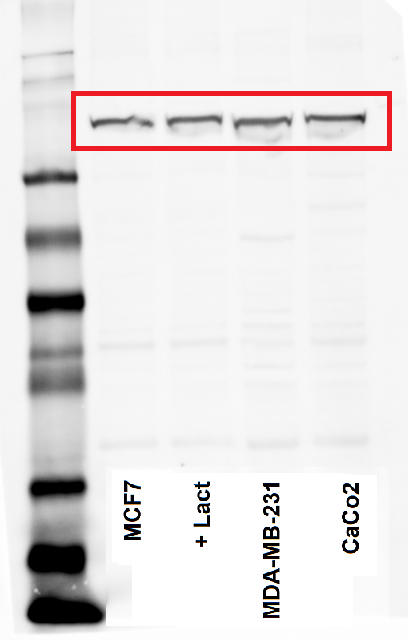

Supplement: Supplementary file 1 [file cancers-15-03728-s001.zip › REVISED Uncropped WB Images/Figure 3/4 cell lines GRP94.tif]

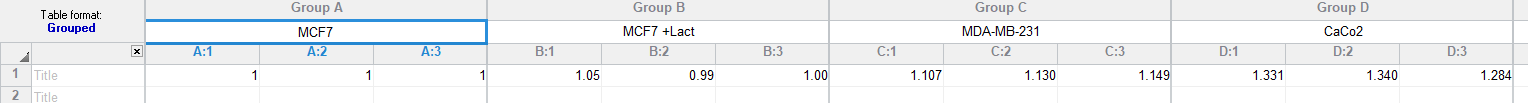

Supplement: Supplementary file 1 [file cancers-15-03728-s001.zip › REVISED Uncropped WB Images/Figure 3/Densitometry GRP94 - 4 cell lines.tif]

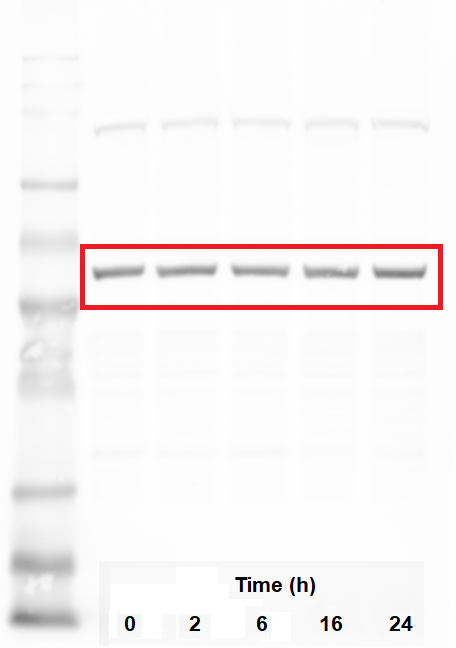

Supplement: Supplementary file 1 [file cancers-15-03728-s001.zip › REVISED Uncropped WB Images/Figure 3/GRP94 in DOXO-exposed cells/CaCo2/CaCo2 doxo ACTIN.tif]

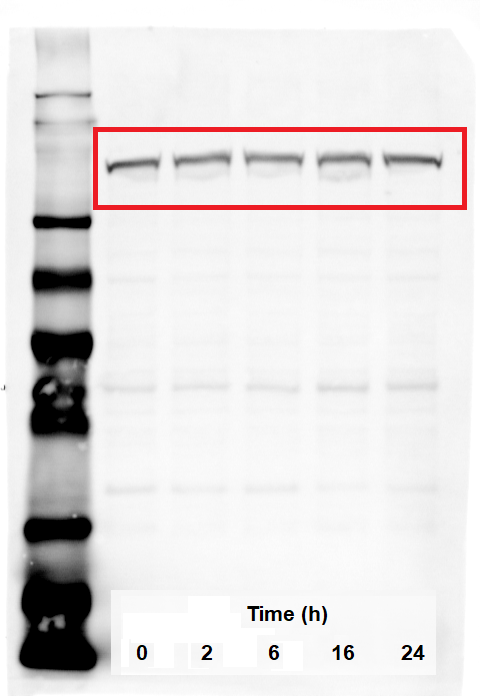

Supplement: Supplementary file 1 [file cancers-15-03728-s001.zip › REVISED Uncropped WB Images/Figure 3/GRP94 in DOXO-exposed cells/CaCo2/CaCo2 doxo GRP94.tif]

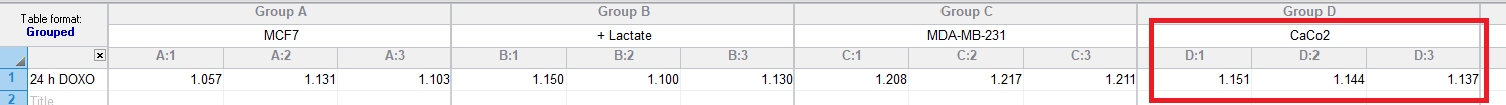

Supplement: Supplementary file 1 [file cancers-15-03728-s001.zip › REVISED Uncropped WB Images/Figure 3/GRP94 in DOXO-exposed cells/CaCo2/Densitometry GRP94 - DOXO 24 h.tif]

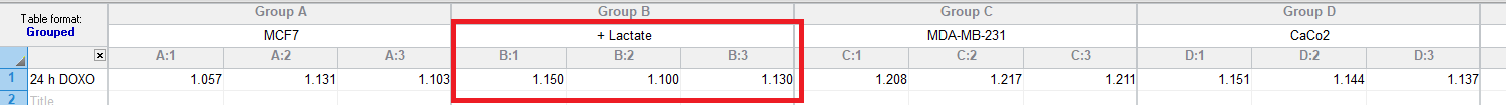

Supplement: Supplementary file 1 [file cancers-15-03728-s001.zip › REVISED Uncropped WB Images/Figure 3/GRP94 in DOXO-exposed cells/MCF7 LACTATE/Densitometry GRP94 - DOXO 24 h.tif]

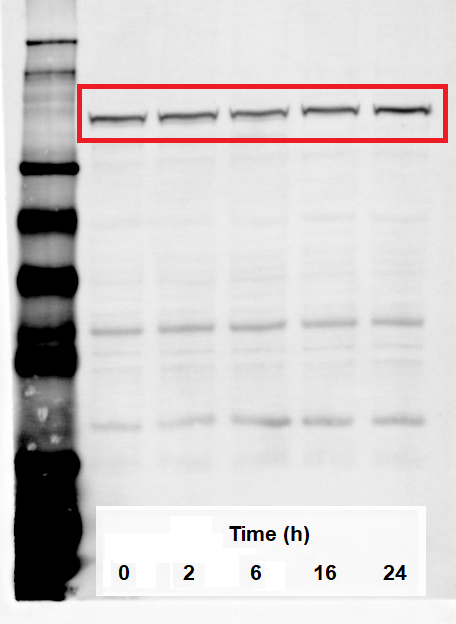

Supplement: Supplementary file 1 [file cancers-15-03728-s001.zip › REVISED Uncropped WB Images/Figure 3/GRP94 in DOXO-exposed cells/MCF7 LACTATE/MCF7 LACT DOXO GRP94.tif]

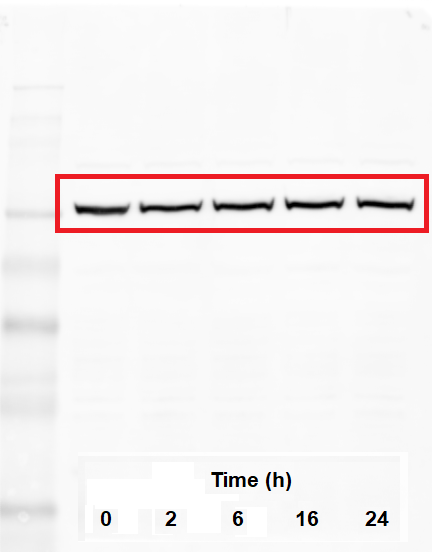

Supplement: Supplementary file 1 [file cancers-15-03728-s001.zip › REVISED Uncropped WB Images/Figure 3/GRP94 in DOXO-exposed cells/MCF7 LACTATE/MCF7 LACT DOXO HSC70 internal standard.tif]

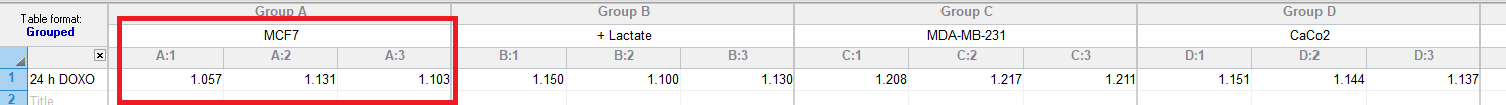

Supplement: Supplementary file 1 [file cancers-15-03728-s001.zip › REVISED Uncropped WB Images/Figure 3/GRP94 in DOXO-exposed cells/MCF7/Densitometry GRP94 - DOXO 24 h.tif]

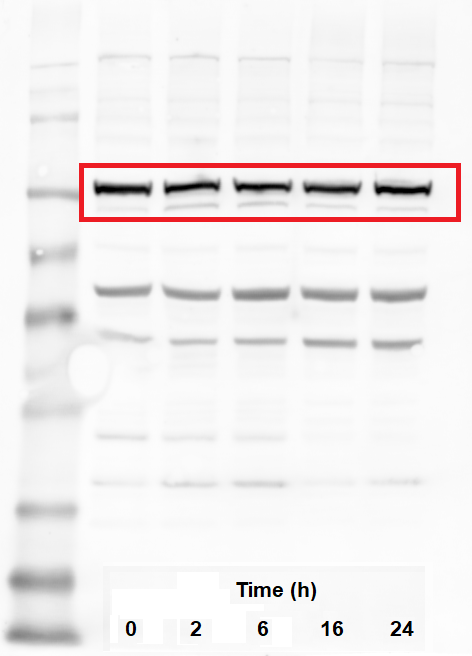

Supplement: Supplementary file 1 [file cancers-15-03728-s001.zip › REVISED Uncropped WB Images/Figure 3/GRP94 in DOXO-exposed cells/MCF7/MCF7 doxo HSC70 internal standard.tif]

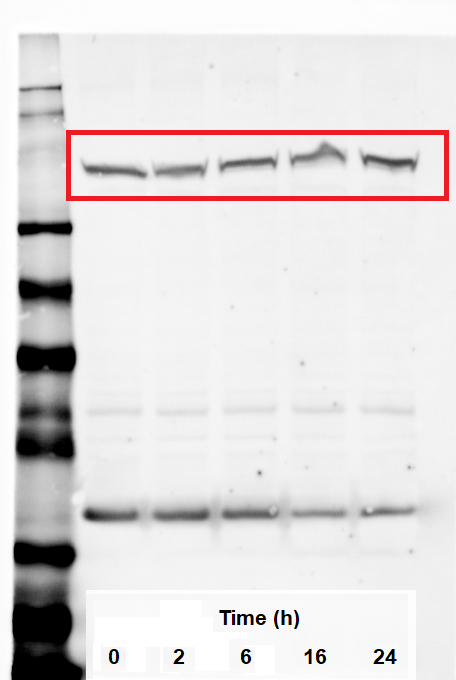

Supplement: Supplementary file 1 [file cancers-15-03728-s001.zip › REVISED Uncropped WB Images/Figure 3/GRP94 in DOXO-exposed cells/MCF7/MFC7 CTR doxo GRP94.tif]

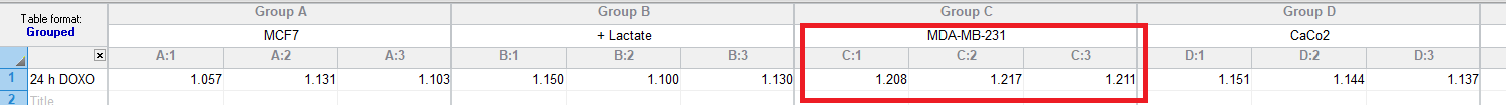

Supplement: Supplementary file 1 [file cancers-15-03728-s001.zip › REVISED Uncropped WB Images/Figure 3/GRP94 in DOXO-exposed cells/MDA-MB-231/Densitometry GRP94 - DOXO 24 h.tif]

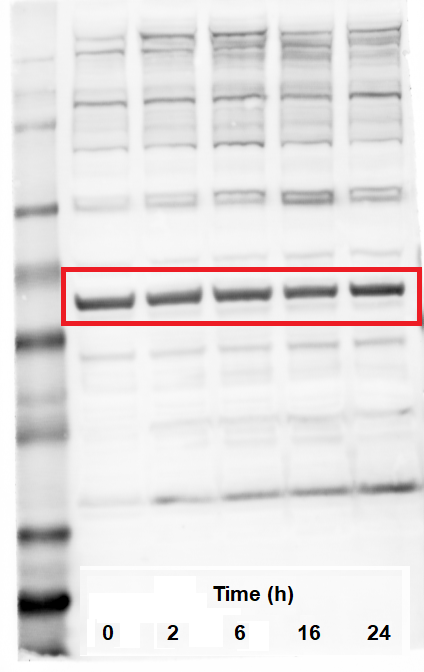

Supplement: Supplementary file 1 [file cancers-15-03728-s001.zip › REVISED Uncropped WB Images/Figure 3/GRP94 in DOXO-exposed cells/MDA-MB-231/MDA-MB-231 doxo ACTIN.tif]

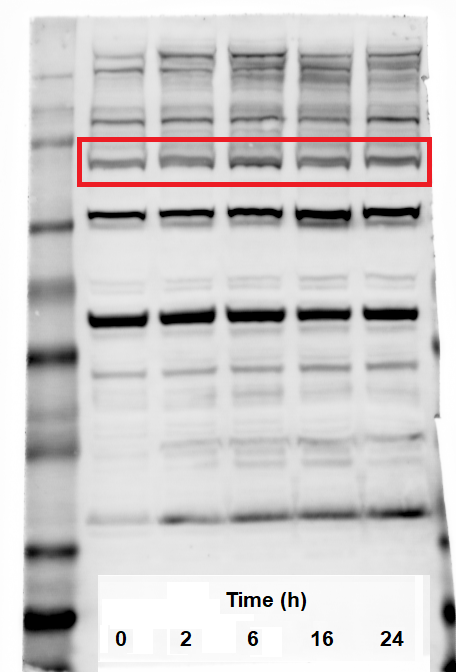

Supplement: Supplementary file 1 [file cancers-15-03728-s001.zip › REVISED Uncropped WB Images/Figure 3/GRP94 in DOXO-exposed cells/MDA-MB-231/MDA-MB-231 doxo GRP94.tif]

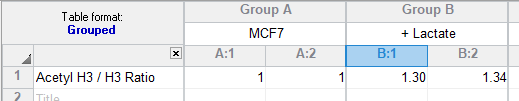

Supplement: Supplementary file 1 [file cancers-15-03728-s001.zip › REVISED Uncropped WB Images/Figure 4/H3/Densitometry H3.tif]

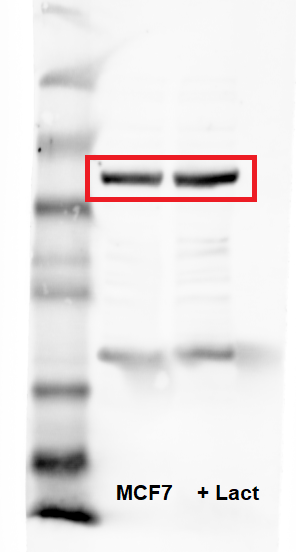

Supplement: Supplementary file 1 [file cancers-15-03728-s001.zip › REVISED Uncropped WB Images/Figure 4/H3/MCF7 H3 ACTIN.tif]

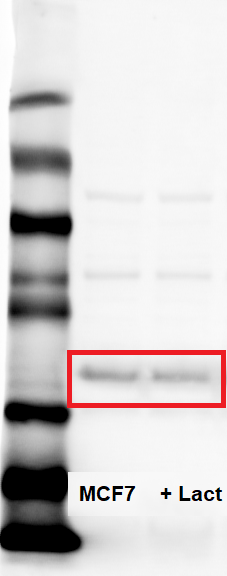

Supplement: Supplementary file 1 [file cancers-15-03728-s001.zip › REVISED Uncropped WB Images/Figure 4/H3/MCF7 H3.tif]

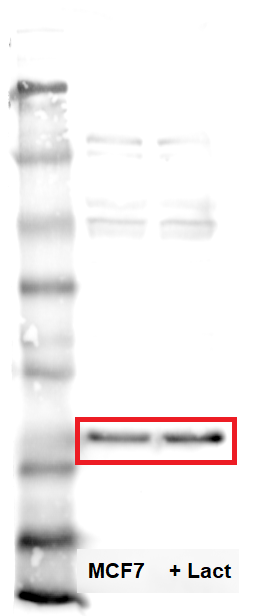

Supplement: Supplementary file 1 [file cancers-15-03728-s001.zip › REVISED Uncropped WB Images/Figure 4/H3/MCF7 pan-acetyl H3.tif]

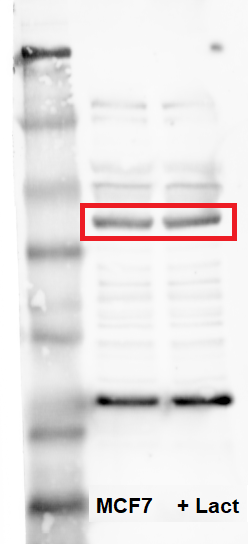

Supplement: Supplementary file 1 [file cancers-15-03728-s001.zip › REVISED Uncropped WB Images/Figure 4/H3/MCF7pan-acetyl H3 ACTIN.tif]

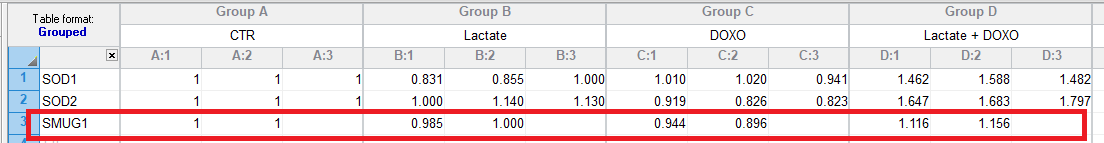

Supplement: Supplementary file 1 [file cancers-15-03728-s001.zip › REVISED Uncropped WB Images/Figure 4/SMUG1/Densitometry SOD1 SOD2 SMUG.tif]

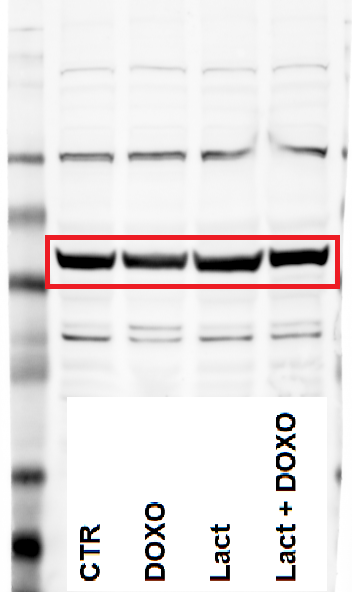

Supplement: Supplementary file 1 [file cancers-15-03728-s001.zip › REVISED Uncropped WB Images/Figure 4/SMUG1/MCF7 doxo 4h smug1 ACTIN.tif]

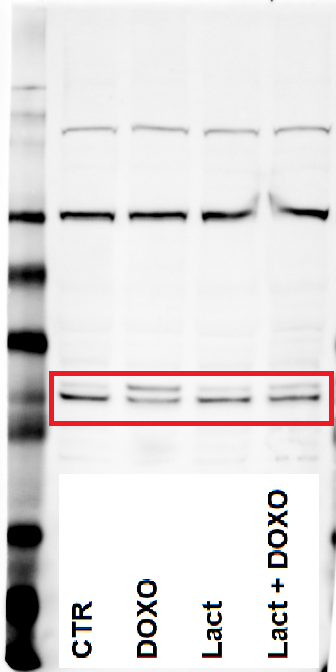

Supplement: Supplementary file 1 [file cancers-15-03728-s001.zip › REVISED Uncropped WB Images/Figure 4/SMUG1/MCF7 DOXO 4h SMUG1.tif]

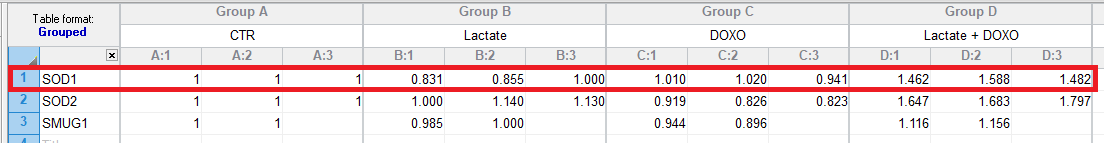

Supplement: Supplementary file 1 [file cancers-15-03728-s001.zip › REVISED Uncropped WB Images/Figure 4/SOD1/Densitometry SOD1 SOD2 SMUG.tif]

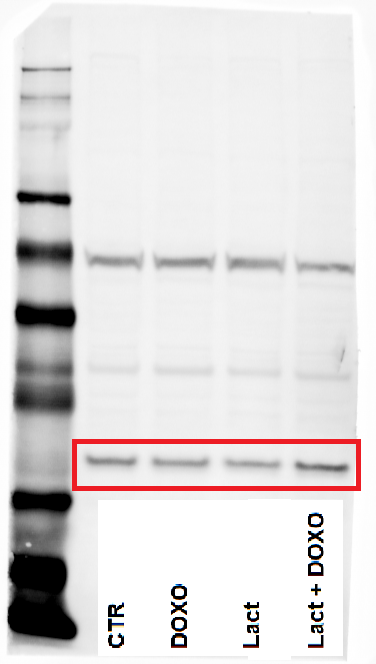

Supplement: Supplementary file 1 [file cancers-15-03728-s001.zip › REVISED Uncropped WB Images/Figure 4/SOD1/MCF7 ctr lact doxo SOD1.tif]

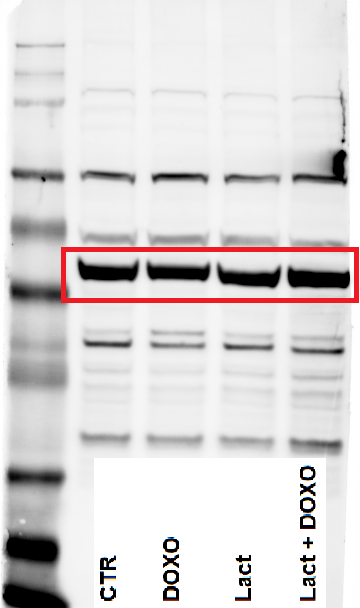

Supplement: Supplementary file 1 [file cancers-15-03728-s001.zip › REVISED Uncropped WB Images/Figure 4/SOD1/MCF7 DOXO 4h sod1 ACTIN.tif]

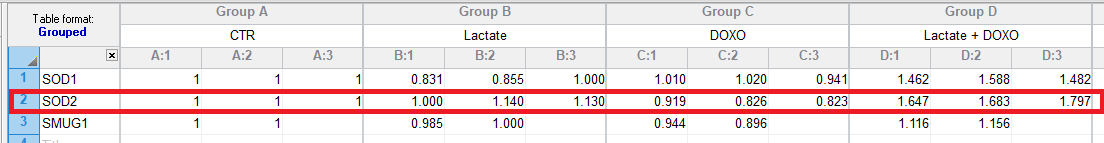

Supplement: Supplementary file 1 [file cancers-15-03728-s001.zip › REVISED Uncropped WB Images/Figure 4/SOD2/Densitometry SOD1 SOD2 SMUG.tif]

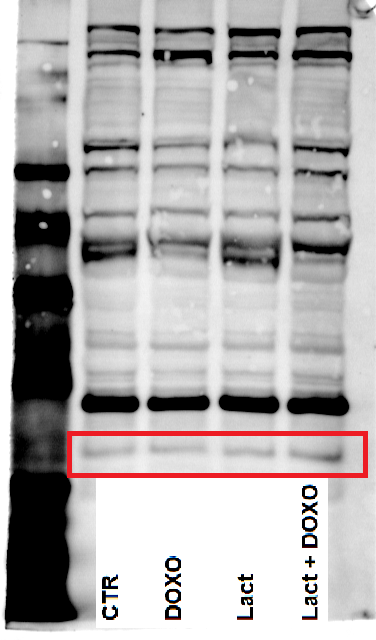

Supplement: Supplementary file 1 [file cancers-15-03728-s001.zip › REVISED Uncropped WB Images/Figure 4/SOD2/MCF7 ctr lact doxo 4h SOD2.tif]

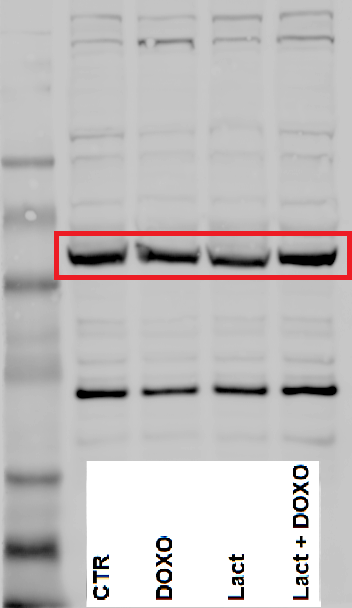

Supplement: Supplementary file 1 [file cancers-15-03728-s001.zip › REVISED Uncropped WB Images/Figure 4/SOD2/MCF7 DOXO 4h sod2 ACTIN.tif]

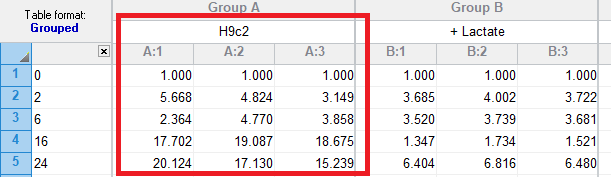

Supplement: Supplementary file 1 [file cancers-15-03728-s001.zip › REVISED Uncropped WB Images/Figure 6/H9c2 CTR/Densitometry H2AX H9c2.tif]

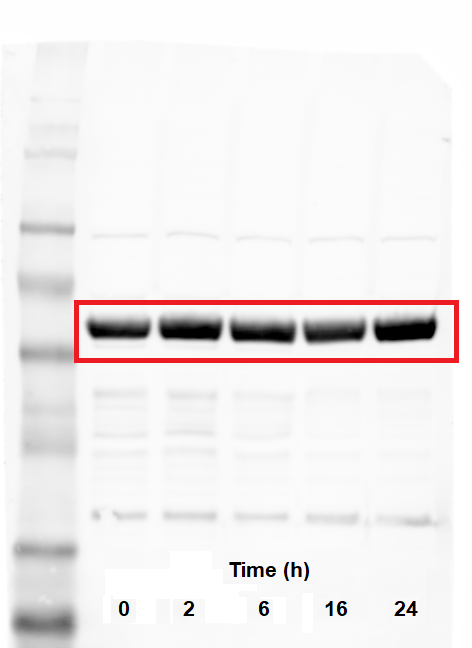

Supplement: Supplementary file 1 [file cancers-15-03728-s001.zip › REVISED Uncropped WB Images/Figure 6/H9c2 CTR/H9c2 ctr doxo ACTIN.tif]

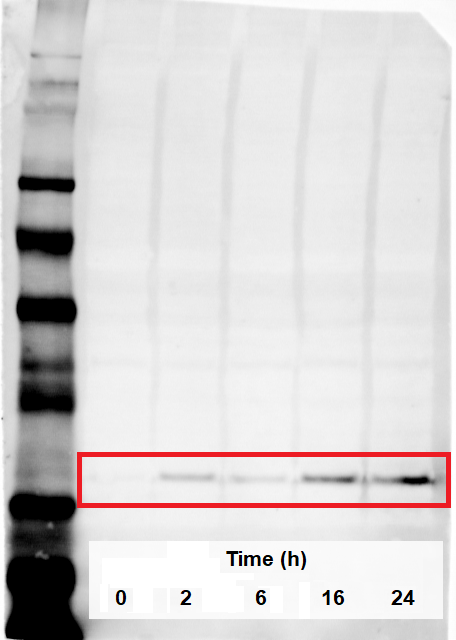

Supplement: Supplementary file 1 [file cancers-15-03728-s001.zip › REVISED Uncropped WB Images/Figure 6/H9c2 CTR/H9c2 ctr doxo H2AX.tif]

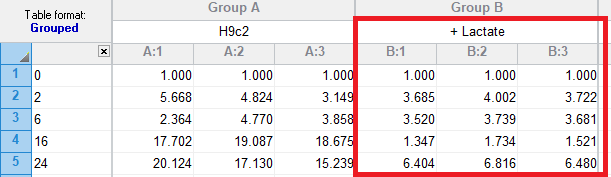

Supplement: Supplementary file 1 [file cancers-15-03728-s001.zip › REVISED Uncropped WB Images/Figure 6/H9c2 LACTATE/Densitometry H2AX H9c2.tif]

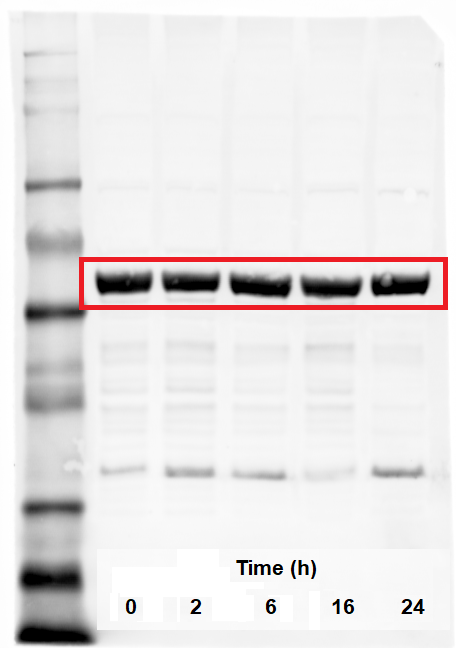

Supplement: Supplementary file 1 [file cancers-15-03728-s001.zip › REVISED Uncropped WB Images/Figure 6/H9c2 LACTATE/H9c2 lactate doxo ACTIN.tif]

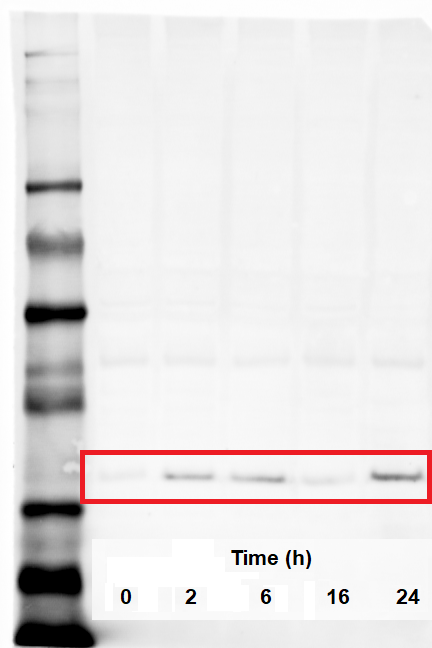

Supplement: Supplementary file 1 [file cancers-15-03728-s001.zip › REVISED Uncropped WB Images/Figure 6/H9c2 LACTATE/H9c2 lactate doxo H2AX.tif]

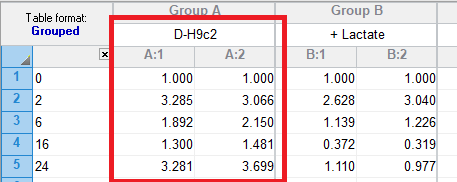

Supplement: Supplementary file 1 [file cancers-15-03728-s001.zip › REVISED Uncropped WB Images/Figure 7/H9c2 CTR/Densitometry H2AX Diff-H9c2.tif]

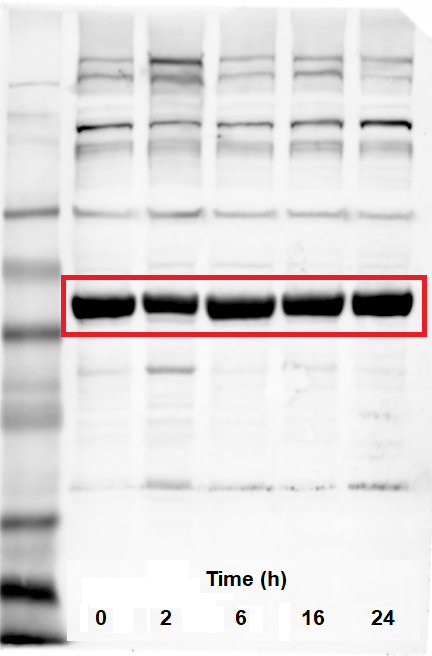

Supplement: Supplementary file 1 [file cancers-15-03728-s001.zip › REVISED Uncropped WB Images/Figure 7/H9c2 CTR/D-H9c2 CTR H2AX ACTIN.tif]

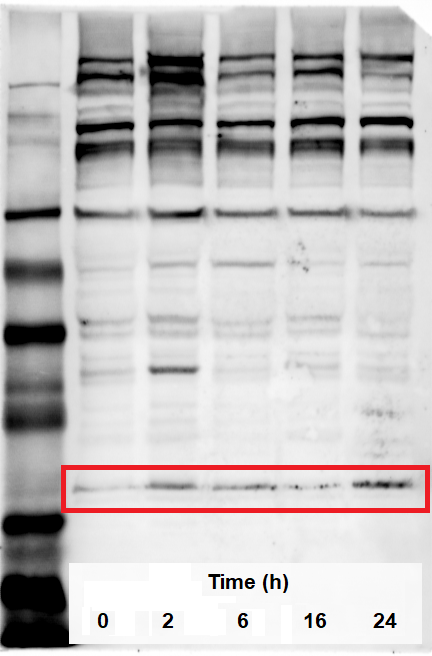

Supplement: Supplementary file 1 [file cancers-15-03728-s001.zip › REVISED Uncropped WB Images/Figure 7/H9c2 CTR/D-H9C2 CTR doxo H2AX.tif]

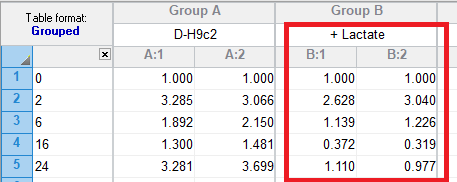

Supplement: Supplementary file 1 [file cancers-15-03728-s001.zip › REVISED Uncropped WB Images/Figure 7/H9c2 LACTATE/Densitometry H2AX Diff-H9c2.tif]

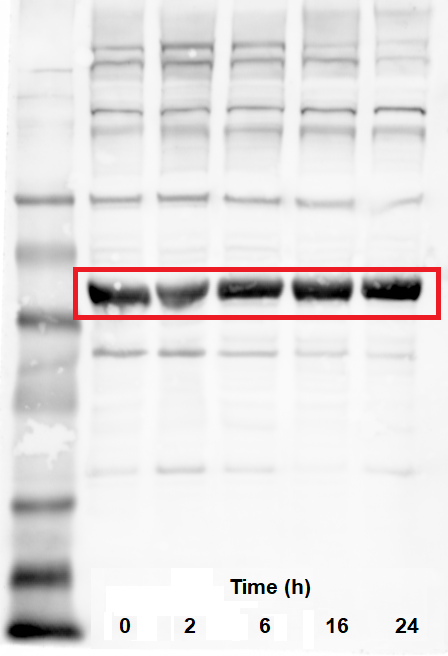

Supplement: Supplementary file 1 [file cancers-15-03728-s001.zip › REVISED Uncropped WB Images/Figure 7/H9c2 LACTATE/D-H9c2 lactate H2AX ACTIN.tif]

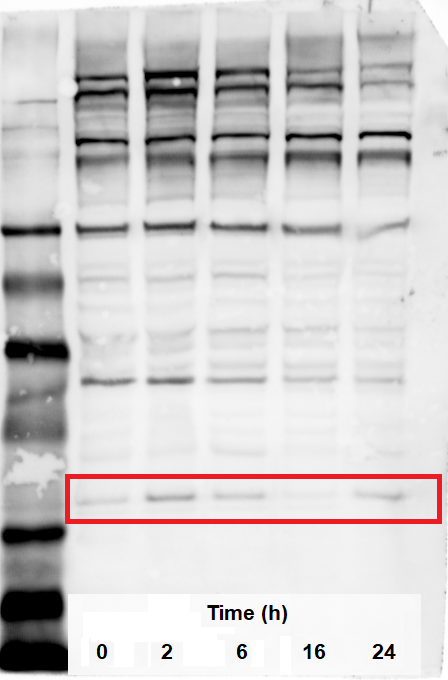

Supplement: Supplementary file 1 [file cancers-15-03728-s001.zip › REVISED Uncropped WB Images/Figure 7/H9c2 LACTATE/D-H9c2 lactate H2AX.tif]

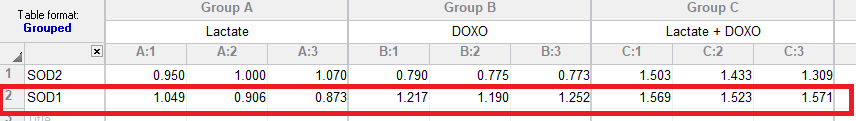

Supplement: Supplementary file 1 [file cancers-15-03728-s001.zip › REVISED Uncropped WB Images/Figure 8/D-H9c2/Densitometry SOD1.tif]

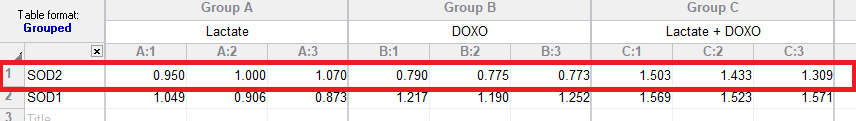

Supplement: Supplementary file 1 [file cancers-15-03728-s001.zip › REVISED Uncropped WB Images/Figure 8/D-H9c2/Densitometry SOD2.tif]

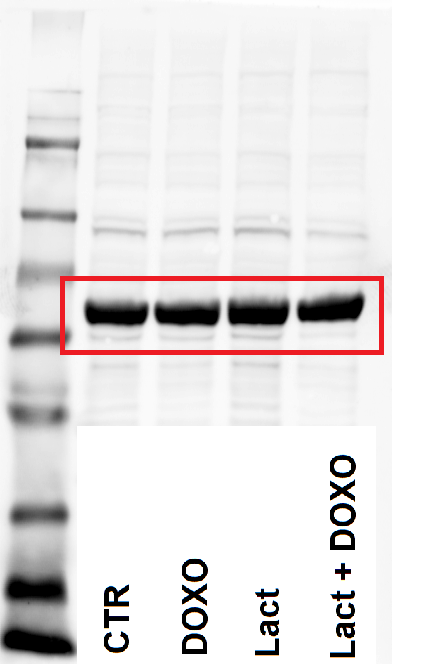

Supplement: Supplementary file 1 [file cancers-15-03728-s001.zip › REVISED Uncropped WB Images/Figure 8/D-H9c2/D-H9c2 ACTIN in SOD1.tif]

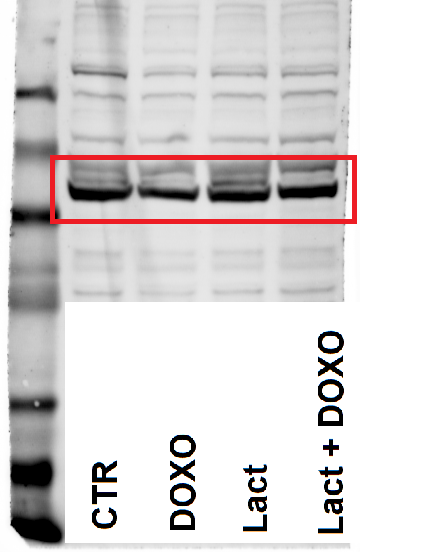

Supplement: Supplementary file 1 [file cancers-15-03728-s001.zip › REVISED Uncropped WB Images/Figure 8/D-H9c2/D-H9c2 ACTIN in SOD2.tif]

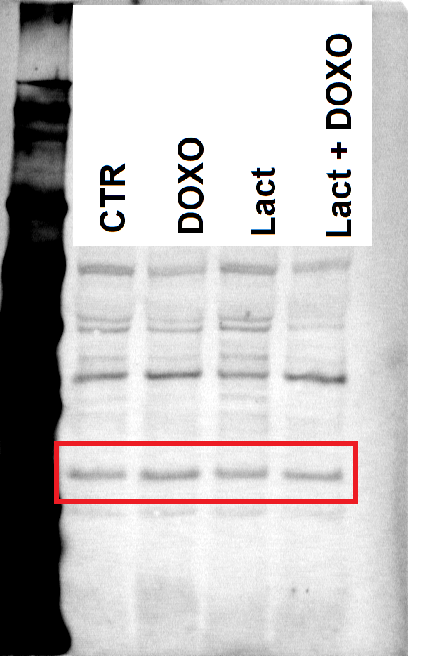

Supplement: Supplementary file 1 [file cancers-15-03728-s001.zip › REVISED Uncropped WB Images/Figure 8/D-H9c2/D-H9c2 SOD1.tif]

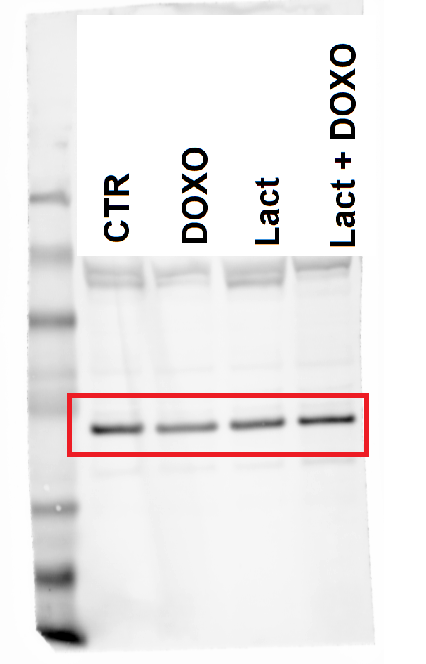

Supplement: Supplementary file 1 [file cancers-15-03728-s001.zip › REVISED Uncropped WB Images/Figure 8/D-H9c2/D-H9c2 SOD2.tif]

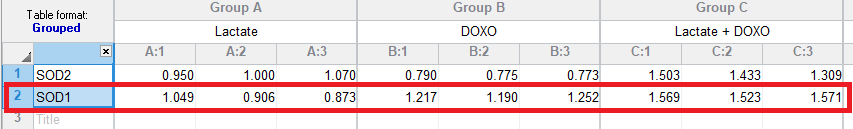

Supplement: Supplementary file 1 [file cancers-15-03728-s001.zip › REVISED Uncropped WB Images/Figure 8/H9c2/Densitometry SOD1.tif]

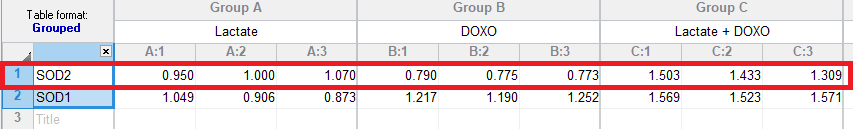

Supplement: Supplementary file 1 [file cancers-15-03728-s001.zip › REVISED Uncropped WB Images/Figure 8/H9c2/Densitometry SOD2.tif]

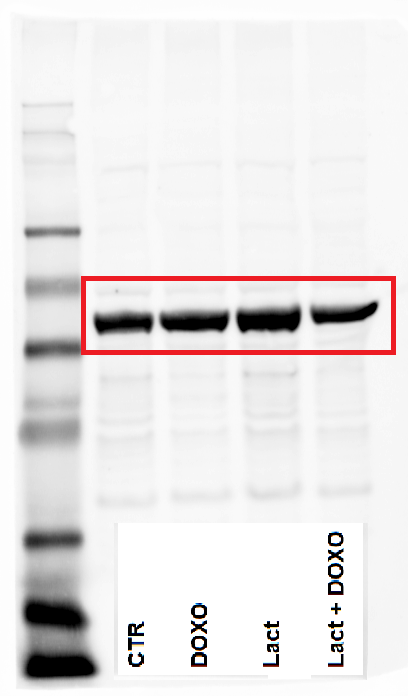

Supplement: Supplementary file 1 [file cancers-15-03728-s001.zip › REVISED Uncropped WB Images/Figure 8/H9c2/SOD1 ACTIN.tif]

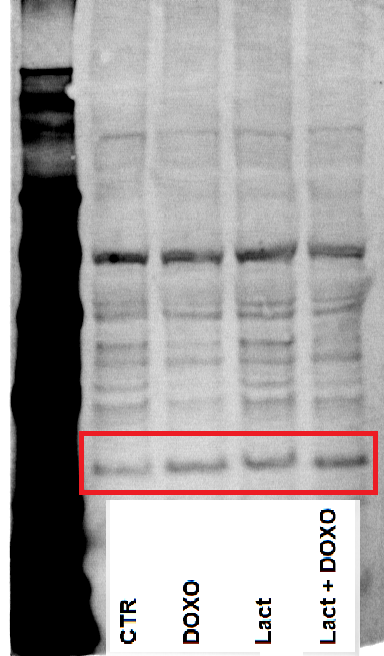

Supplement: Supplementary file 1 [file cancers-15-03728-s001.zip › REVISED Uncropped WB Images/Figure 8/H9c2/SOD1.tif]

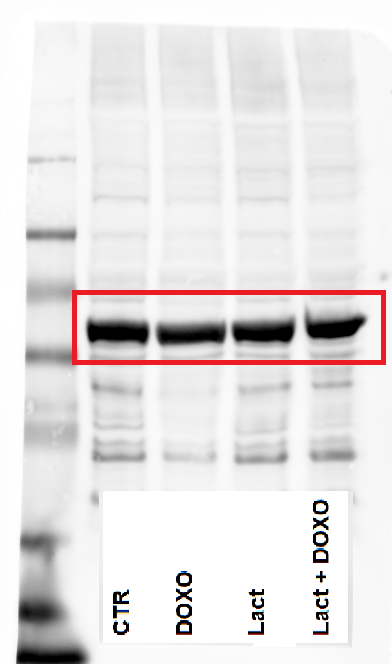

Supplement: Supplementary file 1 [file cancers-15-03728-s001.zip › REVISED Uncropped WB Images/Figure 8/H9c2/SOD2 ACTIN.tif]

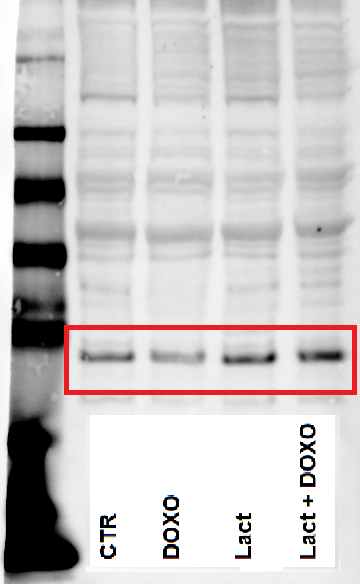

Supplement: Supplementary file 1 [file cancers-15-03728-s001.zip › REVISED Uncropped WB Images/Figure 8/H9c2/SOD2.tif]
